# Cases from the Surgical Clinic of Atlanta Medical College

**Published:** 1871

**Authors:** 


### *Cases from the Surgical Clinic of Atlanta Medical College.*

Dr. W. F. Westmoreland, in charge.

[Reported by J. T. Johnson, M.D.]

L. W., white man, æt 22 ; married ; laborer.

History : Has had a sore on the penis for three months ; has not been treated either locally or constitutionally ; has not changed its character or appearance in that time ; is now hard at the base, elevated, oval in shape, and three or four lines in diameter.

Diagnosis : Hard or Hunterian Chancre, well marked ; in other words, true syphyilis. As further proof of its syphilitic or constitutional character, he has an enlargement just commencing of the lymphatic glands of the posterior cervical region ; in addition, has pharyngitis, and incipient ulceration of the mucous membrane of the mouth.

Remarks : This is syphilis, syphilis proper, as distinguished from the "soft chancre" (now called chancroid), which is altogether local in its nature, and therefore requires only local treatment for its cure. For eighteen years has not treated chancroid, or soft chancre, otherwise than by local means, and has never, in even one single instance, seen result any constitutional or "secondary" manifestations. Chancroid or "soft chancre" is not syphilis, and it is a misnomer to so call it. But in *syphilis* or "hard chancre," such as the case now considered, the disease is constitutional, and the local sore but a manifestation of that general disease of the system. The sore rarely makes its appearance till three or four weeks after contagion.

The disease, then, of this patient, being one of the constitution, requires for its cure remedies addressed to the system at large ; by this means, the chancre will disappear without applying to it any local remedy whatever. The chancre having existed for so long a time without treatment, and without change in character, is well calculated to prove the correctness of these views, and the efficacy of the remedies administered for its cure.

TREATMENT :—℞

Calomel, grs. xij.
Pulv. opii gr. iii.
Mix—divide in 12 powders.
Give one morning and night.

No local application.

24th. No appreciable change in his condition.

28th. Chancre somewhat diminished in size, and presents an improved appearance.

Mercury withheld, as slight soreness of the teeth and gums indicates its impress on the system.

℞—Iodide potassium ℥ss.
Pure water, ℥iv.
Make solution.
Give one table-spoonful three times a day.

*July 1st.*—Chancre has entirely healed. Gums but slightly if at all sore; in consequence of which the mercurial treatment was resumed in the following form :

℞
Blue mass gr. xxx.
Pulv. opium gr. ii.
Make 6 pills.
Give one morning and night.

Also, continue the Iodide of Potassium.

*June 21st.*—H., colored male ; æt 45 ; laborer.

Sight of one eye almost destroyed by injuries received from a powder explosion, while blasting rock. The pupil is contracted, and the iris apparently bound fast by adhesions. Considerable opacity of the cornea, though some portions of it are transparent. He can barely see any object.

TREATMENT.—The indication is to dilate the pupil as much as possible. This can frequently be accomplished by the use of belladonna, even when the disease has reached this stage—to be used constitutionally, or applied locally, or in both ways. This is a remedy that we should never neglect when there is danger of a permanent contraction of the pupil. It can do no harm, and by its use may preserve the sight that would otherwise be lost. Mercury may be also used for its reputed power of absorbing effused lymph.

℞
Ext. belladonna, gr. ij.
Hydrargyri Chloridi Mitis, gr. iij.
Ft. pil. No. vi.
Take one night and morning.

Also, a belladonna plaster to be worn over the eye.

24th.—Sight improving from enlargement of pupil, thus admitting more rays of light. Treatment continued.

28th.—Improvement of sight continues. Can now distinguish letters of very large type. Treatment continued.

*June* 21*st*.—G., colored female ; æt 22. Was recently relieved by operation of a deformity of the upper eyelid, which had resulted from a wound inflicted by the horn of a cow. The wound was a lacerated one, and an improper union resulted, uniting the integument of one lip or flap, with the conjunctiva of the other. In this way a fissure was produced, resembling that of hare-lip. The mucous membrane in contact with the eye was very greatly thickened ; and not only was the appearance unsightly, but the function of the lid was impaired—indeed, totally destroyed. The abnormal attachments were dissected loose, and proper union effected by the twisted suture and adhesive strips.

To-day the sutures are removed, and the appearance justifies the hope of restoration of the shape and functions of the lid. The thickened mucous membrane will probably be reduced, or "absorbed," from the pressure exerted upon it by the lid, in its natural position. The adhesive plaster is re-applied, lest some accident sever the newly-formed attachments.

*July* 12*th*.—M. B., colored female ; æt 16.

The face has, for some time, been covered with an eruption that produces great disfigurement by the splotches, and is evidently syphilitic in character. Other portions of the body, as chest, arms, thighs, etc., are affected, but not to so marked an extent. At the time she came under treatment ( and more so now), it was impossible to assign it to its precise place in the nosology of the *syphilides*. Has been under treatment for two or three weeks. Blue mass and opium, alternated with iodide of potassium. Such alternation is often preferable to the continuous use of either remedy alone. Under this treatment there is steady improvement.

The lymphatic glands of the cervical region, which were enlarged, at the beginning of the treatment, are reduced in size as evidenced by touch. These glands are almost invariably found thus affected in syphilis ; and this is important, as a means of diagnosis ; for, by it we may often assure ourselves of the existence of the disease, when the patient, either from a desire to deceive, or ignorance of the primary trouble or its nature, denies having ever been infected. But we should not forget that these glands may be, and often are, enlarged from causes other than syphilis. Some neighboring local trouble, may give rise to the same condition. Hence, we must be careful to exclude such causes, lest in our diagnosis we make an error through which we would not only adopt erroneous treatment, but even do injustice to the chastity of parties concerned. These glands in question, are not those under or near the angle of the jaw, and which are often increased in size from any trouble in the mouth, but are situated on either side of the posterior region of the neck, constituting a chain along the anterior or upper border of the trapezius muscle.

Now, a practical question presents itself. With treatment of the other manifestations of this disease, should we expect a disappearance of this enlargement? Such is the case, as a rule ; but the reverse sometimes obtains. Has had under observation a gentleman cured of syphilis 4 years ago ; there has been no trouble whatever since that time, though the glands are still in morbid state that they then assumed. He has, in the meantime, married, and become the father of children that are perfectly healthy and have never presented any evidence of hereditary transmission. This and other such exceptions, teach that while we may generally expect their subsidence, we must not regard the disease as uncured, merely because of their remaining permanent.

